# Peripheral levels of monocytic myeloid‐derived suppressive cells before and after first induction predict relapse and survivals in AML patients

**DOI:** 10.1111/jcmm.17576

**Published:** 2022-10-13

**Authors:** Pierre Peterlin, Camille Debord, Marion Eveillard, Alice Garnier, Amandine Le Bourgeois, Thierry Guillaume, Maxime Jullien, Marie C. Béné, Patrice Chevallier

**Affiliations:** ^1^ Hematology Department Nantes University Hospital Nantes France; ^2^ Hematology Biology Nantes University Hospital Nantes France; ^3^ INSERM UMR1232, CRCINA IRS‐UN University of Nantes Nantes France

**Keywords:** ALL, AML, diagnosis, MDSC, monocytes, survivals

## Abstract

Myeloid Derived Suppressive Cells (MDSC) are capable to suppress innate and adaptive immune responses, thus favouring solid cancer progression. However, little is known about the role of MDSC in acute myeloid leukaemia (AML). In this monocentric prospective study, 73 adult AML patients, eligible for first‐line intensive chemotherapy, were included with the aim to study the influence on long‐term outcomes of peripheral blood (PB) levels of monocytic (M) MDSC (M‐MDSC) assessed by flow cytometry. A percentage of peripheral M‐MDSC higher than 0.55% of leukocytes at diagnosis and a decrease of M‐MDSC% after induction came out both as independent negative prognostic factors for leukaemia‐free and overall survival.

## 1 INTRODUCTION

The notion of MDSC encompasses a complex series of cell subsets with the capacity to suppress innate and adaptive immune responses.^1^ In 2016, divergences in their immunophenotypic definition led to propose a harmonization/standardization of strategies to establish a robust enumeration of MDSC subsets. Thus, in humans, M‐MDSC can be characterized in flow cytometry by the minimal CD14^+^/CD11b^+^/CD33^+^/HLA‐DR^−/low^ immunophenotypic pattern, at variance from granulocytic MDSC that lack CD14 and express bright CD15.^2^ The latter are readily identifiable in mice through the Gr marker but this differentiation antigen is lost on human suppressive neutrophils. These cells can be identified in the low‐density fraction of leukocytes after Ficoll Hypaque density centrifugation of human PB, which is a time‐consuming technique difficult to standardize.^2^

MDSC pertain to the physiological mechanisms allowing to regulate and tamper immune responses. As T‐regs or B‐regs, they are used by tumour cells to favour their escape from the normally very efficient anti‐tumoral immune responses. MDSC are also capable of directly inhibiting T‐cell functions. This immunosuppressive action in the setting of malignancy is thought to be due to the secretion by tumour cells and MDSC themselves of various anti‐inflammatory cytokines such as TGF‐β and IL‐10 and many other molecules (arginases, NO, ROS, IDO, PGE2).^3^, ^4^

The pejorative role of MDSC in favouring tumour expansion, described in solid tumours,^5^ has also been reported in various haematological malignancies.^6^ Conversely, MDSC can successfully protect against acute and chronic graft‐versus‐host disease in the context of allogeneic haematopoietic stem cell transplantation (Allo‐HSCT). This is the case when such cells are found in high proportion in the graft infused or in the recipient's PB after transplant.^7^

However, the role of MDSC remains unclear in the setting of AML. From available data, a higher proportion of MDSC, especially M‐MDSC, can be found in patients' PB and bone marrow (BM)^8^, ^9^, ^10^ and could be associated with conventional prognostic factors, response to chemotherapy and minimal residual disease levels.^9^ Yet, their impact on long‐term outcomes has not yet been fully explored, since only one study, to our knowledge, has reported an impact of circulating M‐MDSC, defined as CD14^+^/HLA‐DR^low/−^, showing lesser complete remission rate and overall survival in patients with more than >0.5% M‐MDSC.^10^

## 2 MATERIALS AND METHODS

The monocentric prospective study reported here included all consecutive adult AML patients who received a standard first‐line 3 + 7 intensive chemotherapy in the Haematology Clinic of Nantes University Hospital between October 2017 and March 2021. The main objective was to investigate the presence of PB M‐MDSC at diagnosis and after induction and to correlate their levels with complete cytologic remission (CR) or CR with incomplete platelet recovery (CRi) according to current criteria (ELN2017 classification), leukaemia‐free (LFS) and overall (OS) survivals.

**FIGURE 1:**
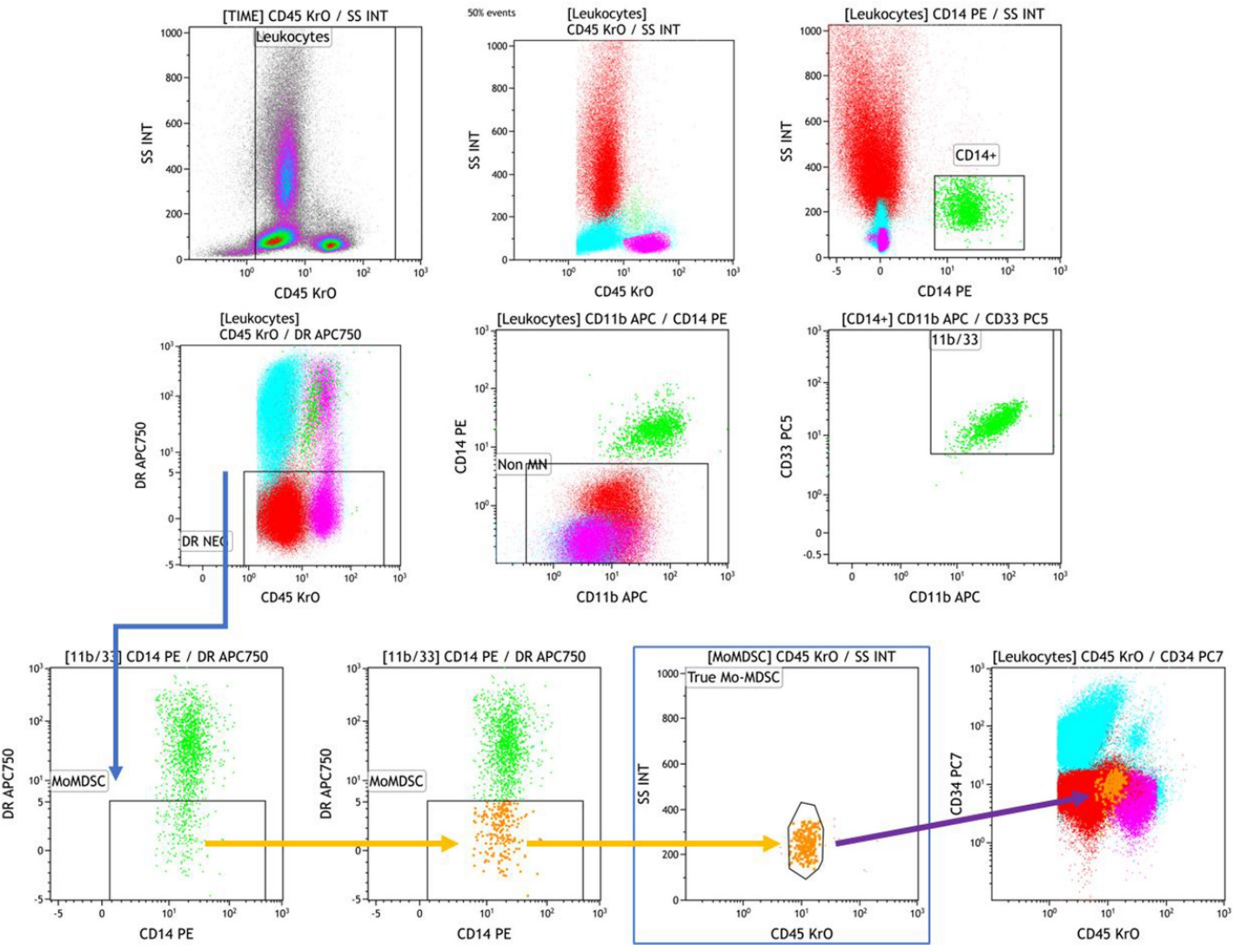
Gating strategy for the identification of Monocytic MDSC (M‐MDSC), in a diagnosis sample with peripheral blasts (cyan, CD45int, DR+, CD34+). *Top row*: density plot (left) and coloured representation (middle, CD45/SSC, red = neutrophils, magenta = lymphocytes, green = monocytes, cyan = blasts) of all leukocyte populations with further segregation of monocytes (CD14^+^, highlighted, right). *Middle row*: definition of the DR‐NEG threshold based on neutrophils and lymphocytes fluorescence intensity (left). Further definition of the monocytic population with CD11b and CD33. *Lower row*: identification of M‐MDSC applying the same threshold for DR‐NEG cells (blue arrow, linked gates). Highlighting of M‐MDSC as rare events (middle) and further definition based on CD45/SSC –(right) (orange arrows). Final backgating on a CD45:SSC scattergram of all leukocytes clearly segregating the blasts from the M‐MDSC population located between neutrophils and lymphocytes (purple arrow)

This study was also performed with the objective of proposing a simple and readily transposable M‐MDSC assay. To this avail, a lysis‐no‐wash flow cytometry technique was developed. After determining the optimal antibody combination and titers, a specific ready‐to‐use tube (Duraclone®) was ordered from Beckman Coulter/Immunotech (Marseilles, France) as described in Table S1. For each assay, 100 μl of PB collected on EDTA were added in a Duraclone® tube, vortexed and incubated in the dark at room temperature for 15 min. Red blood cells were then lysed with 2 ml Versalyse® (Beckman Coulter, Miami, FL) for 15 min in the same conditions. Data acquisition was performed immediately on a Navios® flow cytometer (Beckman Coulter). Analyses used the Kaluza® software (Beckman Coulter) with a dedicated protocol applied to all samples. M‐MDSC were expressed both as a percentage (%) of total nucleated cells defined as CD45+ (Figure 1) and as an absolute count (AC) based on the whole blood cell count of the day of analysis. Patient M‐MDSC% and AC were compared to those of 21 healthy controls (females *n* = 17, median age: 52 years old, range: 39–63).

The study was registered at the French Commission Nationale de l'Informatique et des Libertés as CNIL 2016–038 and approved by the Ethic Review Board of Nantes University Hospital. All patients provided informed consent.

For statistical analyses, M‐MDSC% and AC were considered continuous variables. Comparisons were performed using Mann Whitney tests and survivals were estimated by the LogRank test and Kaplan Meier representation with the R software via BiostaTGV and Medcalc (Ostend, Belgium) software. *p* values lower than 0.05 were considered statistically significant.

## 3 RESULTS

Seventy‐three AML patients were enrolled (Table 1 and Table S2). The median M‐MDSC% at diagnosis was 0.19% (range: 0–54) similar to the median % found in controls (0.24%, 0.02–1.21, *p* = 0.94). M‐MDSC levels were available after chemotherapy induction for 60 patients at a median of 37,5 days (range: 25–89). At that time, the median M‐MDSC% in patients was significantly higher (0.87%, range: 0–28) than in controls (*p* = 0.001) and at diagnosis (*p* = 0.001) Table 2.

**TABLE 1:** Patient characteristics

Characteristics	Acute Myeloid Leukaemia *n* = 73
Median age: years (range)	62 (20–73)
Median follow‐up: months (range)	21 (3–41.5)
Gender: Male/Female	47/26
ELN 2017	
Favourable	12
Intermediate	29
Unfavourable	32
Median WBC at diagnosis: 10^9^/L (range)	4.2 (0.5–236)
Median % blasts in PB	17% (0–99)
Median % blasts in BM	52.5 (20–97)
Allograft during follow up	45 (62%)
CR/CRp	61 (83.5%)
Relapse from CR/CRp	15/61 (24.5%)
Death (*n*)	26
Causes of death	
Relapse (*n*)	20
Infections (*n*)	3
Cerebral haemorrhage (*n*)	2
Cardiac toxicity (*n*)	1
2‐year LFS	52.4+/−6%
2‐year OS	53.6+/−7%

Abbreviations: BM, bone marrow; CR, complete remission; CRp, CR with incomplete platelets recovery; ELN, European Leukaemia Network; LFS, leukaemia‐free survival; OS, overall survival; PB, peripheral blood; WBC, white blood counts.

**TABLE 2:** Univariate analysis

*N* = 73	2‐year LFS	2‐year OS
Gender Female *n* = 26	61.2+/−10% vs	62.9+/−10% vs
Male *n* = 47	48.5+/−8% *p* = 0.14	49.6+/−9% *p* = 0.17
Age < 62yo* *n* = 35	56+/−9% vs	61+/−9% vs
Vs ≥ 62yo *n* = 38	49.2+/−10% *p* = 0.68	51.3+/−10% *p* = 0.61
ELN 2017 Favourable *n* = 12	90+/−9% vs	90+/−9% vs
Int *n* = 29	55.5+/−11% vs	55.5+/−11% vs
Unfavourable *n* = 32	31.3+/−10% *p* = 0.0012	31.3+/−10% *p* = 0.009
WBC < *n* = 36	54.9+/−9% vs	58.9+/−9% vs
vs ≥ median *n* = 37	50.6+/−9% *p* = 0.30	52.2+/−10% *p* = 0.66
%PB blasts < median = 36	55.4 + −9% vs	58 + −9%
vs ≥ median *n* = 36	50 + −11% *p* = 0.70	vs 51.8 + −9% *p* = 0.77
% BM blasts** < median = 35	52.4+/−9% vs	55.7+/−9% vs
vs ≥ median *n* = 35	51.3+/−10% *p* = 0.71	55.5+/−10% *p* = 0.81
M‐MDSC at diagnosis:		
% < 0.55% *n* = 45	67.7+/−8%	71.5+/−8%
vs % ≥ 0.55% *n* = 28	30.1+/−10%, *p* = 0.005	30.1+/−10%, *p* = 0.001
AC <0.012 Giga/L *n* = 36	59.4 + −9%	63.3 + −10%
vs ≥0.012 Giga/L *n* = 37	45.8 + −9%, *p* = 0.22	44.5 + −10%, *p* = 0.07
M‐MDSC post‐induction		
% < 0.87% *n* = 30	52.6 + −11%	53.4 + −10%
vs % ≥ 0.87% *n* = 30	59 + −10%, *p* = 0.68	63.9 + −10%, *p* = 0.49
AC <0.045 Giga/L *n* = 30	54.5 + −10%	58.3 + −10%
vs ≥0.045 Giga/L *n* = 30	55.8 + −11%, *p* = 0.84	58.5 + −10%, *p* = 0.96

Abbreviations: %, percentage; AC, absolute count; BM, bone marrow; ELN, European Leukaemia Network; LFS, leukaemia‐free survival; M‐MDCS, monocytic Monocytic Myeloid‐Derived Suppressive Cells; OS, overall survival; PB, peripheral blood; WBC, white blood count; yo, years old.

^*^all <60 years old **3 patients data not available.

**FIGURE 2:**
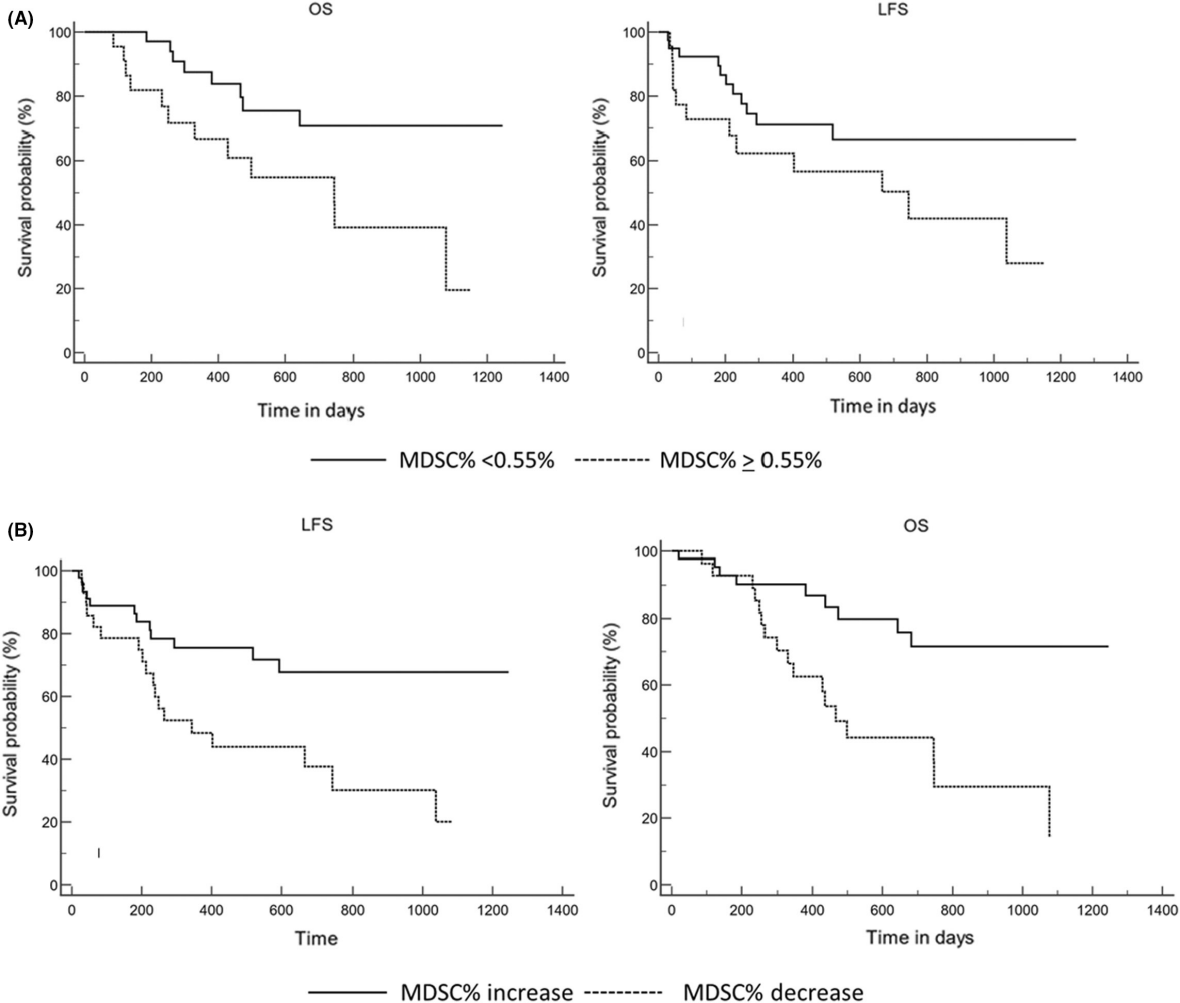
Comparison of A. LFS (left) and OS (right) in AML patients with M‐MDSC% < (full line) or ≥0.55% (dotted line) at diagnosis; B. LFS (left) and OS (right) in AML patients with increasing (full line) M‐MDSC% versus others (dotted line)

Diagnosis M‐MDSC% did not correlate with any other factor, especially ELN2017 classification (*p* = 0.79), the FAB classification (M4/M5 vs others, *p* = 0.34) nor the percentage of peripheral blasts (< vs > median, *p* = 0.50). ROC curves for LFS established the threshold of 0.55% of M‐MDSC at diagnosis as the best cut‐off for analyses. Indeed, 2‐year LFS (67.7 ± 8% vs. 30.1 ± 10%, *p* = 0.005) and 2 years OS (71.5 ± 8% vs. 30.1 ± 10%, *p* = 0.001) (Figure 2A) were significantly higher for patients with low M‐MDSC levels (<0.55%) at diagnosis. Although this threshold was not predictive of CR/CRi (86.6% *n* = 39/45 vs. 78.5% *n* = 22/28, *p* = 0.56), the incidence of cytologic relapse after achieving CR/CRi was significantly lower in these patients (12.8% *n* = 5/39 vs. 45.4% *n* = 10/22, *p* = 0.01). Median post‐induction M‐MDSC% were similar between patients achieving CR/CRi (0.9%, *n* = 53) vs. others (0.44%, *n* = 8, *p* = 0.34). Post‐induction M‐MDSC% had no impact on relapse or survivals. However, a comparison of M‐MDSC% before and after induction in patients did impact outcome. Indeed, an increase (vs stability or decrease) of M‐MDSC% was associated with better 2y‐OS (72.9 ± 8% vs. 39.8 ± 11%, *p* = 0.005) and a trend for 2y‐LFS (68.5 ± 8% vs. 37.2 ± 11%, *p* = 0.03) by univariate analysis (Figure 2B). The response to induction (CR/CRp) was significantly higher (97.2% vs. 69.5%, *p* = 0.007) for patients whose M‐MDSC% increased, comparing pre‐ and post‐induction values (Figure S1). Finally, there was a trend for higher relapse in patients achieving CR/CRp but with a decrease of M‐MDSC% after induction (50% vs 10.8%, *p* = 0.06).

The median AC of M‐MDSC was 0.012 × 10^9^/L (range: 0–4.45) at diagnosis and 0.0001 × 10^9^/L (0–0.4989) after induction. This did not correlate to any other factors and ROC curves for LFS did not establish a best cut‐off for analyses. Median M‐MDSC AC at diagnosis and after induction (comparing < vs ≥) were not predictive of the CR/CRi rate (86% vs. 81%, *p* = 0.79), relapse (19.3% vs. 30%, *p* = 0.50), nor LFS and OS. Very strikingly, the majority of evaluable patients (54/60, 90%) showed a stability/decrease of M‐MDSC AC between pre and post‐induction. This parameter (increase vs not of M‐MDSC AC between pre and after induction) was thus not predictive of outcomes.

Seventy patients benefited from molecular analysis with the local high throughput sequencing AML minimal panel searching for mutations of *NPM1, FLT3ITD, IDH1, IDH2, ASXL1, RUNX1, TP53* at diagnosis. Thus, 5 groups could be delineated: *NPM1*+ (*n* = 20), *FLT3*+/*NPM1*‐ (*n* = 8), negative (*n* = 17), *ASXL1* and/or *RUNX1* and/or *TP53* (*n* = 14), isolated IDH2+ (*n* = 7). Four patients were excluded as they did not display any of these mutations but others had been determined on another extended panel. Only the *ASXL1/RUNX1/TP53* high‐risk group showed a median M‐MDSC% >0.55% before induction. However, when considering this subgroup vs all others, the percentage of patients with a M‐MDSC% ≤0.55% was not statistically different (43% vs. 71%, *p* = 0.09). All molecular groups showed an increase in the median M‐MDSC% after induction. (Table S3).

Multivariate analysis (MA) was performed including age, blast count, ELN17 classification, WBC, sex, M‐MDSC%. This confirmed the independent prognostic value of M‐MDCS% at diagnosis (LFS *p* = 0.02, HR 3.6, 95%CI: 1.88–6.91; OS *p* = 0.02, HR 2.6, 95%CI: 1.11–5.95), the ELN 2017 classification being the only other prognostic factor for survivals (LFS *p* = 0.0001, HR 2.34, 95%CI: 1.10–4.97; OS *p* = 0.01, HR 2.57, 95%CI: 1.18–4.11). When adding the increase or decrease of M‐MDSC% between before and after induction, MA retained the independent prognostic value of M‐MDSC increase on OS (*p* = 0.02, HR 0.34, 95%CI: 0.14–0.85) together with ELN17 classification (*p* = 0.0007, HR 3.94, 95%CI: 1.78–8.69). For LFS, ELN17 classification (*p* = 0.0001, HR 5.75, 95%CI: 2.4–13.76), WBC (*p* = 0.0021, HR 4.3, 95%CI: 1.70–10.9) and MDSC% (*p* = 0.001, HR 3.32, 95%CI: 1.32–8.36) were retained, but not M‐MDSC variation.

## 4 DISCUSSION

This study thus seems to demonstrate a strong correlation between a higher percentage of peripheral M‐MDSC at diagnosis (>0.55% of total nucleated cells) and worse outcomes, especially LFS (as shown by multivariate analysis), in adult AML patients not previously treated and receiving a standard 3 + 7 intensive induction chemotherapy. This result confirms what has been reported also for various solid tumours.^5^ Conversely, in AML, only one study of 62 cases has reported a negative impact of M‐MDSC% at diagnosis both on the CR rate and OS, yet with a different definition of M‐MDSC and no notion of the threshold used. In that study, survival curves seemed rather reflect OS between patients achieving CR vs those who did not.^10^

The study reported here seems also to demonstrate a predominant and independent role of M‐MDSC proportions among leukocytes at AML diagnosis. Of note, the percentage but not the absolute value of M‐MDSC was associated with prognosis. This recalls what has been published for the blast decrease rate during induction, where the percentage, i.e. individual relative proportion of the cells of interest among leukocytes, carried significance.^11^

These results sustain that the immunosuppressive properties of MDSC at diagnosis can disadvantage the host immune system in its fight against AML cells. Various mechanisms have been already reported to explain these properties. Indeed, high V‐domain Ig suppressor of T‐cell activation (VISTA) expression has been documented on MDSC with the consequence to inhibit CD8+ T cells responses in this setting.^12^ Also, AML blasts alter the immune microenvironment through enhanced arginine metabolism probably implicating MDSC.^13^ Other mechanisms implying AML‐derived extracellular vesicles may both induce monocytes to acquire an MDSC phenotype^14^ and enhance MDSC proliferation via MUC‐1.^15^

Surprisingly, the median M‐MDSC% at diagnosis was not higher compared to controls, as reported in the literature for AML.^8^, ^9^ However, it must be noted that this control population was not strictly sex and age‐matched.

Intriguingly, the kinetics of peripheral M‐MDSC% showed a significant positive impact on OS of increased post‐induction levels. This suggests that a potential dual role of M‐MDSC has to be considered. Elevated M‐MDSC at diagnosis could favour tumour escape by suppressing immune responses, while M‐MDSC reconstitution after chemotherapy could help to avoid recurrence of the disease and/or deleterious treatment‐related complications. If the role of reconstituted M‐MDSCs remains to be clarified, a change in tumour environment after induction may explain these disparities of M‐MDSC activity. The dual immunosuppressive and not immunosuppressive functions of M‐MDSC of similar immunophenotype and biochemical profile, clearly shown by Bronte et al.^2^ seem to find here clear support.

Targeting MDSC as a therapeutic approach in cancer is increasingly considered. Various MDSC‐inhibiting strategies may be envisioned including a direct attack of MDSC applying such agents as tyrosine kinase, IL‐6R or S100A9 inhibitors, metformin or anti‐CD38 monoclonal antibodies. Other strategies would be to induce MDSC differentiation into mature myeloid cells through the use, for example, of vitamins A, D3 or E or ATRA or to promote MDSC deactivation via down regulation of arginase‐1 or NOS2 expression, these molecules being highly expressed by activated MDSC.^5^, ^6^

Of course, these results need to be validated in an independent cohort, but clinical studies should investigate in the future the combination of such agents with chemotherapy in AML. However, it will be a significant challenge to design combination therapies that target both decrease of M‐MDSC at diagnosis and increase of these cells after induction.

Of note, bone marrow MDCS should probably be explored also, yet keeping in mind that it may be difficult to distinguish between maturing monocyte progenitors and these cells. Of note, in this series and as shown in Figure 1, the AML PB blast population did not overlap with M‐MDSC.

Data are scarce also on the role of MDSC in acute lymphoblastic leukaemia. This study included 14 acute lymphoblastic leukaemia (ALL) patients (median age: 53 years old [19–71], 11 B‐ALL, 3 T‐ALL). Peripheral M‐MDSC% at diagnosis were not predictive of response (13/14 CR/CRp), nor LFS or OS for these patients with a good outcome.

In conclusion, these data seem to demonstrate that elevated PB M‐MDSC% at diagnosis together with an increase of this percentage after induction appear as new independent prognostic biomarkers in AML, that could usefully be applied to the development of novel therapeutic strategies in this setting.

## AUTHOR CONTRIBUTIONS

**Pierre Peterlin:** Conceptualization (equal); investigation (equal); writing – original draft (equal). **Camille Debord:** Formal analysis (equal); visualization (equal). **Marion Eveillard:** Visualization (equal). **Alice Garnier:** Investigation (equal); visualization (equal). **Amandine Le Bourgeois:** Investigation (equal); visualization (equal). **Thierry Guillaume:** Investigation (equal); visualization (equal). **Maxime Jullien:** Investigation (equal); visualization (equal). **Marie C. Bene:** Conceptualization (equal); formal analysis (equal); methodology (equal); validation (equal); writing – review and editing (equal). **Patrice Chevallier:** Conceptualization (lead); funding acquisition (equal); investigation (equal); methodology (equal); supervision (lead); validation (equal); writing – review and editing (equal).

## FUNDING INFORMATION

This work was funded by a grant AOI from Nantes University Hospital ref. DHU Oncogreffe 2016.

## CONFLICTS OF INTEREST

The authors declare no conflict of interest.

## Supporting information


Appendix S1

## Data Availability

The data that supports the findings of this study are available in the supplementary material of this article.
