# Supplementary material for: Peripheral levels of monocytic myeloid‐derived suppressive cells before and after first induction predict relapse and survivals in AML patients
Source: J Cell Mol Med. 2022 Oct 13;26(21):5486–92. doi: 10.1111/jcmm.17576 (PMC9639029; doi:10.1111/jcmm.17576)

**Letter to the Editor**

**Peripheral levels of monocytic myeloid-derived suppressive cells ~~at diagnosis~~ before and after first induction predict relapse and survivals in AML patients.**

Pierre Peterlin,^1^ Camille Debord,^2^ Marion Eveillard,^2^ Alice Garnier,^1^ Amandine Le Bourgeois,^1^ Thierry Guillaume,^1,3^ Maxime Jullien,^1^ Marie C Béné*,^2,3^ Patrice Chevallier,*^1,3^

**Supplemental material** :

**Table S1** : Optimal antibody combination and volumes for M-MDSC characterization.

| **Clone** | 80H5 | RMO52 | D3HL60.251 | 581 | Bear1 | Immu-357.12 | 3G8 | J33 |
| --- | --- | --- | --- | --- | --- | --- | --- | --- |
| **Spec** | CD15 | CD14 | CD33 | CD34 | CD11b | HLA DR | CD16 | CD45 |
| **Dye** | FITC | PE | PC5 | PC7 | APC | APC-A750 | PB | KrO |
| **Volume** | 5µL | 5µL | 2µL | 1µL | 2µL | 1µL | 2µL | 5µL |

| **Table S2** | | | | | | | | | | | | | | | | | |
| --- | --- | --- | --- | --- | --- | --- | --- | --- | --- | --- | --- | --- | --- | --- | --- | --- | --- |
| **#** | **Age** | **Sex** | **FAB** | **Karyotype** | **Molecular data** | **Molecular group** | **ELN 2017** | **WBC**  **10^9^/L** | **BM blasts %** | **PB blasts %** | **M-MDSC % Diag** | **M-MDSC AC diag. 10^9^/L** | **POST IND.** | **M-MDSC % post-ind.** | **Allo SCT** | **RELAPSE** | **DEATH** |
| **1** | 69 | M | UC | CK with KMT2A amp., del(5q), del(7q), del(17) | No mutation | neg | adv | 42,8 | 25 | 40 | 1,78 | 0,7618 | R | 0,33 | No | Yes | Yes |
| **2** | 59 | M | UC | trisomy(8) | *ASXL1* | asxl1+ or runx1+ | adv | 0,5 | 24 | NA | 0 | 0 | CRi | 0,84 | Yes | No | *Yes* |
| **3** | 40 | M | M5-B | NK | *FLT3-ITD* (0,61)*, NPM1* | NPM1+ | int | 22 | 48 | 7 | 9,04 | 1,9888 | CR | 5,82 | Yes | Yes | Yes |
| **4** | 70 | F | M2 | NK | *FLT3-ITD* (1,31)*, NPM1* | NPM1+ | int | 9,6 | 59 | 30 | 1,05 | 0,1008 | CRi | 0,18 | No | Yes | Yes |
| **5** | 71 | F | UC | NK | *FLT3-ITD (0,68), NPM1* | NPM1+ | int | 236 | 94,5 | 52 | 0,02 | 0,0472 | CRi | 4,64 | No | No | No |
| **6** | 37 | M | M0 | monosomy7 and inv(3) MECOM | *SRSF2, NRAS* | other | adv | 10 | 58 | 40 | 1,98 | 0,198 |  | 2,8 | Yes | Yes | Yes |
| **7** | 50 | F | M0 | t(3p;21q), trisomy 8; RUNX1 rearranged | No mutation | neg | adv | 1,3 | 33,5 | 0 | 0,072 | 0,0009 | CR | 0,37 | Yes | No | No |
| **8** | 67 | M | M2 | NK | *IDH2* | IDH2+ | int | 0,95 | 56 | 7 | 0,6 | 0,0057 | CR | 0,71 | Yes | No | No |
| **9** | 43 | M | M1 | del(6p), del(17p) not involving *TP53* | *FLT3-ITD* (2) | flt3+/npm1- | adv | 3,45 | 68 | 40 | 0,04 | 0,0014 | CR | 0,41 | Yes | No | No |
| **10** | 62 | F | M1 | NK | No mutation | neg | int | 1,4 | 46 | 0 | 0 | 0 | CR | 0 | Yes | No | No |
| **11** | 62 | F | M2 | NK | *IDH2* | IDH2+ | int | 0,9 | 54,5 | 0 | 0 | 0 | CR | 0,48 | Yes | No | No |
| **12** | 65 | M | M4 | NK | *ASXL1* | asxl1+ or runx1+ | adv | 41 | 84 | 40 | 10,86 | 4,4526 | R | 0,55 | Yes | Yes | Yes |
| **13** | 64 | M | M1 | NK | *IDH2* | IDH2+ | int | 1,4 | 80 | 67 | 0,1 | 0,0014 | CR | 0,63 | Yes | Yes | Yes |
| **14** | 49 | F | M2 | NK | *FLT3-ITD* (0,64) | flt3+/npm1- | adv | 94 | 81 | 75 | 0,009 | 0,0085 | R | 1,8 | Yes | Yes | Yes |
| **15** | 59 | F | UC | CK, del(5q), monosomy 7 | No mutation | neg | adv | 1,3 | 38,5 | 3 | 2,9 | 0,0377 | CR | 0,81 | Yes | Yes | Yes |
| **16** | 52 | F | M2 | t(8;21) | ND | ND | fav | 14.2 | 65,5 | 41 | 0,09 | 0,0128 | CR | 2,17 | No | No | No |
| **17** | 22 | F | M5 | NC | *NPM1* | NPM1+ | fav | 12 | 52 | 5 | 0,16 | 0,0192 | CR | 1,3 | No | No | No |
| **18** | 70 | H | M2 | trisomy 8 | *NPM1* | NPM1+ | int | 3,3 | 24 | 11 | 0,46 | 0,0152 | R | NA | No | No | Yes |
| **19** | 70 | H | M2 | NK | *CEBPA* (biallelelic) | other | fav | 3,5 | 52 | 52 | 0,29 | 0,0101 | CR | 3,65 | Yes | No | No |
| **20** | 69 | F | UC | NK | ND | ND | int | 1,4 | 21 | 4 | 4,48 | 0,0627 | CR | 0,6 | Yes | No | No |
| **21** | 66 | F | UC | CK, del(5q), monosomy 17 | No mutation | neg | adv | 0,9 | 40 | 16 | 1,22 | 0,0109 | CR | 0,62 | Yes | Yes | Yes |
| **22** | 66 | H | M1 | NK | *NPM1* | NPM1+ | fav | 180 | 97 | 97 | 0,0096 | 0,0173 | CR | NA |  | Yes | Yes |
| **23** | 68 | H | UC | NK | *NPM1* | NPM1+ | fav | 12 | 38 | 70 | 0,05 | 0,006 | CR | 0,25 | No | No | No |
| **24** | 69 | F | M7 | CK, del(5q), monosomy 7, del(17) | No mutation | neg | adv | 3,6 | 20 | 0 | 0,77 | 0,0277 | CR | 1,65 | *Yes* | Yes | Yes |
| **25** | 67 | H | M0 | CK | ND | ND | adv | 11 | 21 | 2 | 0,59 | 0,0649 | CR | 0 | *No* | Yes | Yes |
| **26** | 50 | F | M5 | CK, del(5q), t(9;1,1)(p22;q23) | No mutation | neg | adv | 40 | 40 | 24 | 0,46 | 0,184 | CR | 11,85 | Yes | No | No |
| **27** | 64 | F | M2 | CK | No mutation | neg | adv | 1,6 | 32 | 16 | 1,7 | 0,0272 | NBA | NA | Yes | No | No |
| **28** | 55 | M | UC | NK | *FLT3-TKD, NPM1* | NPM1+ | fav | 20 | 20 | 28 | 4,65 | 0,93 | CR | 0 | No | Yes | Yes |
| **29** | 48 | F | M0 | NK | *ASXL1, RUNX1* | asxl1+ or runx1+ | adv | 1,4 | 75 | 10 | 0 | 0 | R | 0 | Yes | Yes | *Yes* |
| **30** | 47 | F | M4 | NK | *NPM1* | NPM1+ | fav | 17 | 62 | 7 | 0,05 | 0,0085 | CR | NA | Yes | Yes | No |
| **31** | 71 | F | M1 | CK, t(9;11) | No mutation | neg | adv | 7,3 | 72 | 90 | 0 | 0 | R | 0 | No | Yes | Yes |
| **32** | 71 | M | M1 | NK | No mutation | neg | int | 0,9 | 64 | 20 | 0 | 0 | CRi | 2,09 | No | Yes | Yes |
| **33** | 69 | M | UC | NK | *NPM1* | NPM1+ | fav | 2,4 | 35 | 19 | 0 | 0 | CR | 2,38 | No | No | No |
| **34** | 40 | M | M2 | NK | *NPM1* | NPM1+ | fav | 5 | 20 | 7 | 0,21 | 0,0105 | CR | 0 | Yes | No | No |
| **35** | 21 | F | M5-A | t(9;10) | *KRAS, CEBPA*  (monoallelic) | other | int | 2,2 | 82,5 | 0 | 0 | 0 | CR | 2,89 | No | No | No |
| **36** | 30 | F | M2 | NK | *FLT3-ITD* (0,83)*, NPM1* | NPM1+ | int | 4 | 36 | 10 | 0 | 0 | CRi | 0 | No | No | No |
| **37** | 69 | M | M5-B | NK | *FLT3-ITD* (0,69)*, NPM1, DNMT3A* R882 | NPM1+ | int | 28 | 87 | 4 | 2,85 | 0,798 | CR | 0,75 | No | No | No |
| **38** | 70 | M | UC | NK | *FLT3-ITD, NPM1, IDH2, CSF3R* | NPM1+ | int | 0,64 | 73,5 | 0 | 0 | 0 | CR | 0,98 | No | No | No |
| **39** | 70 | M | UC | t(16;16) | No mutation | neg | fav | 15 | 75 | 76 | 0,32 | 0,048 | CR | 0 | No | No | No |
| **40** | 48 | M | M2 | NK | *NPM1* | NPM1+ | fav | 6 | 24 | 55 | 0,16 | 0,0096 | CRi | 1,13 | No | No | No |
| **41** | 49 | M | M4 | NK | *NPM1* | NPM1+ | int | 15 | 24,5 | 8 | 0 | 0 | CR | 0,9 | Yes | Yes | No |
| **42** | 71 | M | M1 | Trisomy 8, t(1;17) | *ASXL1* | asxl1+ or runx1+ | adv | 3,3 | 62 | 24 | 54 | 1,782 | CRi | 28 | No | Yes | Yes |
| **43** | 49 | F | M1 | t(9;11) | No mutation | neg | int | 2,2 | 80 | 38 | 26,19 | 0,5762 | CR | NA | Yes | Yes | Yes |
| **44** | 65 | M | M2 | NK | *FLT3-ITD* (0,25)*, IDH2, RUNX1, SRSF2, NRAS* | asxl1+ or runx1+ | int | 73 | 63 | 60 | 1,47 | 1,0731 | CRi | NA | Yes | No | Yes |
| **45** | 47 | M | M4 | t(6;12), del(12p) | *RUNX1, EZH2* | asxl1+ or  runx1+ | adv | 30 | 80 | 45 | 1,9 | 0,57 | R | 3,18 | Yes | Yes | No |
| **46** | 66 | M | M2 | trisomy 8, -Y | *ASXL1, RUNX1, IDH2, CEBPA* (biallelelic) | asxl1+ or runx1+ | int | 7,5 | 40 | 12 | 1,5 | 0,1125 | CRi | 3,7 | Yes | No | Yes |
| **47** | 71 | F | M4 | NK | *CEBPA* (biallelelic) | other | int | 8,2 | 33 | 18 | 2,9 | 0,2378 | CR | 8,4 | No | No | No |
| **48** | 63 | M | M2 | trisomy 11 | *IDH2, ZRSR2* | IDH2+ | int | 1,5 | 40,5 | 25 | 0,83 | 0,0124 | CR | 1,24 | Yes | No | No |
| **49** | 68 | F | M1 | NK | *IDH2, SRSF2, DNMT3A* | IDH2+ | int | 35 | 91 | 96 | 0,19 | 0,0665 | CR | NA | Yes | No | No |
| **50** | 39 | M | M4 | MECOM, t(3;3), monosomy 7 | No mutation | neg | adv | 4,4 | 24 | 2 | 0 | 0 | R | NA | Yes | Yes | Yes |
| **51** | 66 | F | M1 | NK | *FLT3-ITD* (0,44)*, NPM1, CEBPA* (monoallelic) | NPM1+ | int | 73 | 90 | 28 | 0,04 | 0,0292 | CRi | 21 | No | No | No |
| **52** | 68 | M | M2 | t(16;21)(q24;q22) | *FLT3-ITD, RUNX1, NRAS, SRSF2* | asxl1+ or runx1+ | adv | 2,62 |  | 8 | 0,05 | 0,0013 | CRi | 2,64 | No | No | No |
| **53** | 65 | M | M0 | NK | *IDH2, ASXL1* | asxl1+ or runx1+ | adv | 2,4 | 25 | 0 | 1,5 | 0,036 | CR | 1,3 | Yes | No | No |
| **54** | 49 | M | M4 | NK | *FLT3-ITD* (0,64) | flt3+/npm1- | adv | 12 | 93 | 73 | 0 | 0 | CR | 0,11 | Yes | No | No |
| **55** | 59 | M | UC | monosomy7 | No mutation | neg | adv | 2,2 | 20,5 | 6 | 1,15 | 0,0253 | CR | 2,5 | Yes | O5/10/2020 | Yes |
| **56** | 63 | M | M5B | NK | *NPM1, DNMT3A, TET2* | NPM1+ | fav | 8 | 32,5 | 0 | 0,48 | 0,0384 | CRi | 3,5 | No | No | No |
| **57** | 68 | M | UC | trisomy 8 | *ASXL1, IDH1, ZRSR2, EZH2, TET2, PTPN11.* | asxl1+ or runx1+ | int | 1 | 48 | 0 | 0,7 | 0,007 | CRi | NA | No | Yes | Yes |
| **58** | 50 | M | M5B | NK | *NPM1, IDH2, NRAS* | NPM1+ | int | 20 | 34 | 3 | 0,59 | 0,118 | CR | 1,66 | No | No | No |
| **59** | 35 | F | M5A | t(9;11), hyperdiploidy | No mutation | neg | int | 1,6 | 86 | 17 | 0,44 | 0,0070 | CR | 1,5 | Yes | No | No |
| **60** | 58 | M | UC | Trisomy 8 et monosomy 7 | *FLT3-TKD* | flt3+/npm1- | adv | 8 | 30,5 | 9 | 0,75 | 0,06 | R | 0,15 | Yes | Yes | Yes |
| **61** | 25 | M | M1 | Trisomy 6 | *No mutation* | neg | int | 11,7 | 89 | 65 | 0 | 0 | CR | NA | Yes | No | No |
| **62** | 62 | M | M2 | NK | *ASXL1, RUNX1, BCOR, STAG2, PTPN11* | asxl1+ or runx1+ | adv | 1,9 | 26 | 0 | 0,98 | 0,0186 | CR | 0,16 | Yes | No | No |
| **63** | 43 | M | M0 | NK | *RUNX1, FLT3-TKD, SRSF2, STAG2, BCORL1* | asxl1+ or runx1+ | adv | 4 | 72 | 39 | 0 | 0 | CR | 0,56 | Yes | No | No |
| **64** | 52 | M | M1 | NK | *FLT3-ITD* | flt3+/npm1- | adv | 3,67 | 60 | 48 | 0 | 0 | CR | 0,45 | Yes | No | No |
| **65** | 48 | M | M0 | NK | *IDH2, DNMT3A* | IDH2+ | int | 2,1 | 53 | 2 | 0,1 | 0,0021 | CR | 0,67 | Yes | No | No |
| **66** | 21 | M | M1 | monosomy 7 | *FLT3-ITD, IDH2, CEBPA* (biallelelic) | flt3+/npm1- | adv | 160 | 93 | 95 | 0,34 | 0,544 | CR | 0,17 | Yes | No | No |
| **67** | 34 | F | M2 | NK | *RUNX1, NRAS, BCOR, BCORL1, EZH2* | asxl1+ or runx1+ | adv | 2 | 36 | 3 | 0,16 | 0,0032 | CR | 0,3 | Yes | Yes | No |
| **68** | 66 | F | M1 | trisomy 11 | *IDH2* | IDH2+ | int | 20 | 84 | 99 | 0 | 0 | CR | 0 | Yes | No | No |
| **69** | 66 | M | M2 | NK | *RUNX1, SMC1A, SF3B1* | asxl1+ or runx1+ | adv | 3 | 21 | 1 | 0,55 | 0,0165 | CR | 2,38 | Yes | No | No |
| **70** | 20 | M | UC | monosomy 7 | *Flt3-ITD, NRAS, WT1* | flt3+/npm1- | adv | 109 | 53 | 95 | 0 | 0 | R | NA | Yes | Yes | No |
| **71** | 59 | M | M2 | tetrasomy 8 | *FLT3-ITD, IDH2, SRSF2* | flt3+/npm1- | int | 2,2 | 67 | 51 | 0,09 | 0,0019 | CR | 1,45 | Yes | Yes | No |
| **72** | 73 | M | M2 | - Y | No mutation | neg | adv | 2,2 | 25 | 0 | 0,09 | 0,0019 | CR | 2,32 | Yes | No | No |
| **73** | 57 | M | M5A | CK | *NPM1* | NPM1+ | adv | 76 | 86 | 77 | 0,18 | 0,1368 | CR | NA | Yes | No | No |

Legend= WBC : white blood cells; AC: absolute count; UC: unclassifiable; NK: normal karyotype; CK: complex karyotype; *FLT3-ITD* ratio indicated in brackets when available; fav: favorable ; int: intermediate ; adv: adverse; R: refractory; CR: Compete Remission; CRi: Compete Remission with incomplete hematological recovery (CRi); NA: not available, ND: not done.

**Table S3** : % of M-MDSC according to several molecular groups.

| Molecular groups | Median M-MDSC% before Induction n=66 | Median M-MDSC% after Induction n=54 |
| --- | --- | --- |
| NPM1+/FLT3- | 0.16% (0-9.04) n=20 | 1.05% (0-5.82) n=16 |
| FLT3+/NPM1- | 0.02%(0-0.75) n=8 | 0.41% (0.11-1.81) n=7 |
| ASXL1/RUNX1/Tp53 | 0.84% (0-54) n=14 | 1.07% (0-28) n=12 |
| negative | 0.44% (0-26.19) n=17 | 0.81% (0-11.85) n=13 |
| IDH2+ ((without NPM1/FLT3/ASXL1/RUNX1/TP53 | 0.1 (0-0.83) n=7 | 0.65% (0-1.124) n=6 |

**Figure S1** : Individual evolution of M-MDSCs % and absolute counts (AC) before and after induction in non-responders/relapsing (A and C) and responders (B and D).


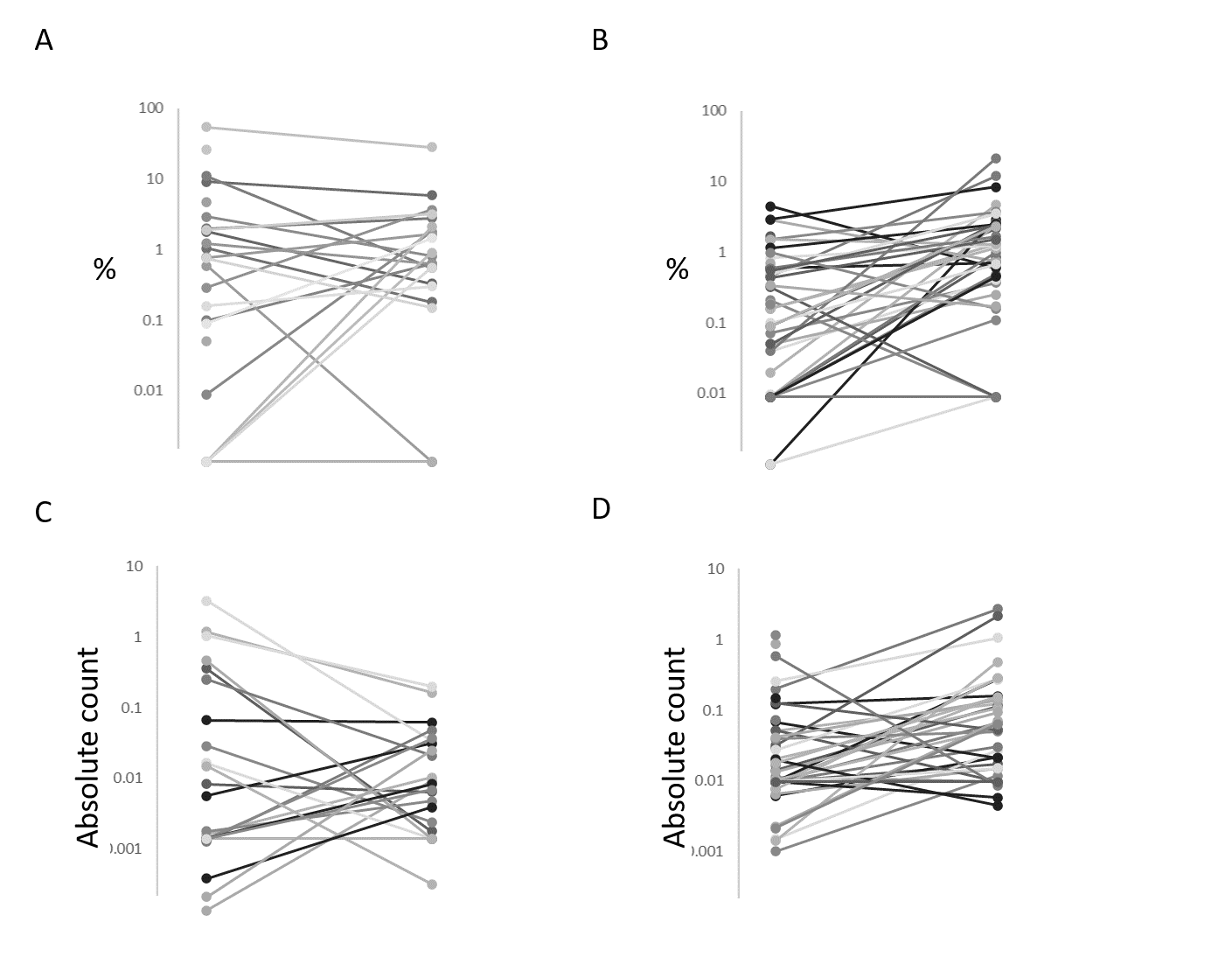

Supplement: Supplementary file 1 — Appendix S1 [file JCMM-26-5486-s001.docx]
